# Sources of Microtemporal Clustering in Sociolinguistic Sequences

**DOI:** 10.3389/frai.2019.00010

**Published:** 2019-06-20

**Authors:** Meredith Tamminga

**Affiliations:** Department of Linguistics, University of Pennsylvania, Philadelphia, PA, United States

**Keywords:** sociolinguistics, persistence, priming, style-shifting, simulation, corpus

## Abstract

Persistence is the tendency of speakers to repeat the choice of sociolinguistic variant they have recently made in conversational speech. A longstanding debate is whether this tendency toward repetitiveness reflects the direct influence of one outcome on the next instance of the variable, which I call sequential dependence, or the shared influence of shifting contextual factors on proximal instances of the variable, which I call baseline deflection. I propose that these distinct types of clustering make different predictions for sequences of variable observations that are longer than the typical prime-target pairs of typical corpus persistence studies. In corpus ING data from conversational speech, I show that there are two effects to be accounted for: an effect of how many times the /ing/ variant occurs in the 2, 3, or 4-token sequence prior to the target (regardless of order), and an effect of whether the immediately prior (1-back) token was /ing/. I then build a series of simulations involving Bernoulli trials at sequences of different probabilities that incorporate either a sequential dependence mechanism, a baseline deflection mechanism, or both. I argue that the model incorporating both baseline deflection and sequential dependence is best able to produce simulated data that shares the relevant properties of the corpus data, which is an encouraging outcome because we have independent reasons to expect both baseline deflection and sequential dependence to exist. I conclude that this exploratory analysis of longer sociolinguistic sequences reflects a promising direction for future research on the mechanisms involved in the production of sociolinguistic variation.

## 1. Introduction

Quantitative sociolinguists have long known that in conversational speech, speakers tend to repeat the choice of the sociolinguistic variant they have recently made. Following Szmrecsanyi (2006), I call this phenomenon *persistence*^1^. Persistence has been observed for a wide range of variables across multiple languages, including pronominal alternations in Quebec French (Sankoff and Laberge, 1978), the passive alternation in English (Weiner and Labov, 1983; Estival, 1985), /s/-deletion and /n/-deletion in Puerto Rican Spanish (Poplack, 1980, 1984), verbal /s/ omission in some varieties of English (Poplack and Tagliamonte, 1989), /s/-deletion in Brazilian Portuguese (Scherre and Naro, 1991, 1992; Scherre, 2001), the English dative alternation (Gries, 2005), particle placement in English (Gries, 2005; Szmrecsanyi, 2006), English coronal stop deletion (Tamminga, 2016), and more. The evidence is abundant that a speaker's choice of variant for a variable at any given moment is partly predictable from their most recent variant choice for the same variable.

How to explain this phenomenon, though, is more controversial. Broadly speaking, there are two classes of explanation. Tamminga et al. differentiate between *sequential dependence*, which is when “the outcome of a sociolinguistic alternation in one moment directly influences the likelihood of a matching outcome some moments later” (Tamminga et al., 2016, p. 33), and *baseline deflection*, which is when “two closely-proximal instances of a sociolinguistic variable are more likely to occur under similar social-contextual circumstances than two instances that are further apart, and thus are more likely to have matching outcomes” (Tamminga et al., 2016, p. 34). Both of these could in principle produce the kind of microtemporal clustering that has been called persistence. Research on persistence sometimes assumes sequential dependence and attributes the dependence to priming, in the psycholinguistic sense of facilitated access to a recently encountered linguistic form ^2^. But it has also been repeatedly observed that stylistic forces might produce apparently similar repetitiveness. To trace an example in the literature, Weiner and Labov (1983) find that speakers are more likely to choose a passive construction instead of an active one when they have already recently used a passive. Weiner and Labov attribute this to both a “mechanical tendency to preserve parallel structure” (suggesting sequential dependence) and “a stylistic factor operating” (suggesting baseline deflection) (Weiner and Labov, 1983, p. 56). In a subsequent study building on Weiner and Labov's results, Estival concludes that “the effect we have been studying [is] a syntactic priming effect” (Estival, 1985, p. 21). In other words, she asserts that persistence in the passive involves sequential dependence in the form of structural priming. On the other hand, Branigan et al. raise the possibility of baseline deflection when they point out that Weiner and Labov's result “might just reflect shifts in the register used during the interviews which they studied” (Branigan et al., 1995, p. 492). Distinguishing between these possibilities is not straightforward.

In this paper I propose that we can make some progress in disentangling sequential dependence and baseline deflection by looking at sequences of multiple observations of the variable prior to a target instance of that variable, instead of just the immediately prior observation. These sequences reflect a string of prior instances on which the speaker had to choose between two^3^ variants of the same sociolinguistic variable as in the target, each of which may be separated by some distance from the target and from other prior observations. For a variable with two possible variants A and B, the usual approach to persistence is to ask whether the probability of choosing B at target T is different based on whether the prior token was A or B: does the outcome in what I will call the A-T and B-T conditions differ?^4^ If we extend our view back to the *two* choices the speaker made before the target, it will give us four conditions: A-A-T, B-A-T, A-B-T, and B-B-T ^5^. I call this a 2-prior sequence, and say that the B-A-T sequence has a 1-back variant of A and a 2-back variant of B (that is, I use “2-prior” to refer to the total depth of the sequence before the target, and “2-back” to refer to a single observation in a particular position within the sequence). We can then ask how the probability of getting B at the target T differs in those four conditions. For instance, we might hypothesize that the observed rate of B in the target will be higher in the A-B-T condition than the B-A-T condition because in A-B-T, the prior instance of B occupies a slot closer in the sequence to the target.

In section 2.3, I conduct this type of quantitative analysis on 2-prior, 3-prior, and 4-prior sequences for the variable ING^6^ in conversational speech. ING is the alternation between the velar and alveolar nasal after unstressed /ɪ/, as in *working* vs. *workin'*. Previous work has attributed ING persistence to priming (Abramowicz, 2007; Tamminga, 2014, 2016), but this variable has also been shown to exhibit style-shifting within data very comparable to that used here (Labov, 2001), making ING a suitable test case for this analysis. Both in section 2.3 and in further statistical analyses of the corpus data in section 2.4, I will demonstrate that the probability of the /ing/^7^ variant is influenced by how many instances of /ing/ occur in the N-prior sequence, as well as by which variant occurs in the 1-back position. There is not, however, evidence that the probability of /ing/ in the target additionally depends on the ordering of the variants at a depth greater than 1-back.

After showing how N-prior sequences influence ING outcomes in the corpus data, I turn in section 3 to a series of simulations to explore what kind of process may have produced the patterns observed in speech. I create a series of simulations based on Bernoulli processes—in essence, modeling sociolinguistic variation as the flipping of weighted coins. The simulations can be set up to have different sources of microtemporal clustering built in, or to exclude such sources. One version of the simulation has sequential dependence built in, while others involve various simple versions of baseline deflection. With each simulation, I generate a dataset that can be analyzed using the same approach as I took with the corpus data, allowing for an intuitive comparison of the outcomes. While every simulation with any source of microtemporal clustering built in produces a difference of some magnitude based on the 1-prior sequence (that is, the analog to the usual persistence effect), the predicted probability as a function of the 3-prior sequence can differ more substantially between models containing baseline deflection and ones containing sequential dependence.

The possibilities for this type of simulation are enormous, and pursuing an exhaustive search of what it might produce is beyond the scope of such preliminary work as this paper. I will, however, suggest that each of the two mechanisms of microtemporal clustering maps more cleanly and consistently to one of the two central effects in the corpus data: baseline deflection can produce the effect of how many times /ing/ occurred in the prior sequence and sequential dependence straightforwardly gives rise to the effect of the immediately-prior token. The pattern seen in the corpus ING data, then, can be produced most effectively by a simulation in which I include both sequential dependence and baseline deflection mechanisms. I argue that this is a welcome result because there are independent reasons to believe in linguistic behavioral phenomena (as I discuss in the following subsection) that should give rise to both of these types of clustering. Finding out that their combination is necessary to produce observed microtemporal patterns in corpus data suggests that future work on persistence might move beyond either/or questions about the source of persistence.

### 1.1. A Terminological Note

The sociolinguistics and corpus linguistics literatures have often used the term “priming” for persistence. Objections to this designation have usually been framed in terms of “style-shifting” or “register changes.” I will avoid using these terms throughout this paper even though the discussion would surely read more intuitively if I contrasted “priming” (sequential dependence) models with “style-shifting” (baseline deflection) models. However, I will maintain that the content- and context-blind quantitative modeling I will explore in this paper does not and cannot distinguish between different real-world interpretations of the microtemporal structures I am exploring. It is tempting to suggest that sequential dependence should be interpreted as the psychological effect of priming—which would itself still leave many questions about the priming mechanism unanswered. However, stylistic and discourse-structural considerations could also give rise to an effect of true sequential dependence. For instance, even if a choice of a particular word order alternant was made purely stochastically, unrelated to contextual preferences, a speaker might wish to continue with the same choice on later utterances in order to maintain the parallelism of the discourse. Similarly, speakers might tend toward repetitiveness itself as a stylistic choice rather than making a series of independent choices that happen to all be occurring under the influence of the same external situation. The same ambiguity is present when it comes to baseline deflection. It may seem most natural to understand shifts in a speaker's target variant rate as being the result of style-shifting, but it is also quite possible to think of psychological factors that could have a similar effect in jointly shaping sequences of target outputs. For instance, a speaker might be operating under a greater memory or attentional burden at some stretches of speech than others, which in turn might influence self-monitoring behavior. The quantitative approach taken here does not distinguish these possibilities; it only distinguishes between the quantitative properties of baseline deflection and sequential dependence. The evidence for how these distinct sources of microtemporal clustering should be interpreted will have to come from other directions. Most importantly, the evidence on this question of interpretation will need to come from conversational corpus data analysis that attends to speaker identity and behavior in particular sociointeractional contexts; such work might conceivably be supplemented by focused, socially sensitive experimental investigations.

## 2. Prior Sequences of the ING Variable

In previous work, I have shown that there is a relationship between a token of ING and the most recent token of ING from the same speaker (Tamminga, 2014, 2016), specifically that the speaker is likely to repeat their immediately prior variant choice. This is consistent with earlier work from Abramowicz (2007), as well as with the corpus persistence literature more generally. Here I use the same underlying dataset as in my previous work to extend my consideration of ING persistence to 2-prior, 3-prior, and 4-prior sequences.

### 2.1. Data

The conversational speech data come from the Philadelphia Neighborhood Corpus (PNC, Labov and Rosenfelder, 2011). The PNC contains sociolinguistic interviews recorded in Philadelphia between 1972 and 2012. The recordings have been orthographically transcribed, then automatically forced-aligned at the word and phone level using the FAVE-align component of the FAVE suite (Rosenfelder et al., 2011). The master ING dataset used here, which comes from a 118-speaker subset of the PNC, is the same as that described in Tamminga (2014, 2016); more detail on the speaker demographics can be found there. To create that dataset, I coded all of the ING observations in the sample auditorily using a Praat script to facilitate exhaustive searching of the corpus' FAVE-aligned TextGrids ^8^. The data are coded with 0 representing /in/ and 1 representing /ing/, so values closer to 1 indicate a higher probability of the /ing/ variant being chosen. The data used for analysis in the current paper is a subset of this master ING dataset; details of how and why this particular subset was chosen are given in section 2.2 below. The primary predictor of interest in this study is the makeup of the N-prior sequence. Each ING token was coded for the values of the four prior ING observations from the same speaker, modulo the exclusions described in section 2.2. The multivariate analyses described in section 2.4 also include the following control predictors:

- Whole word frequency: the Lg10CD measure from SUBTLEX (Brysbaert and New, 2009)
- Speech rate: the number of vowels per second in a 7-word window centered on the target word, which is automatically collected by the Praat script originally used to code the data
- Preceding coronal: in this dataset ING shows progressive dissimilation
- Following pause: in this dataset /ing/ is more frequent before a pause
- Speaker gender: male or female, since ING is a classic stable variable, with women on average using more /ing/ than men.

### 2.2. Revisiting the Envelope of Variation

In quantitative sociolinguistics, deciding what to count and how to count it is a crucial process, sometimes called defining the envelope of variation. I give special attention to these decisions here because, as I point out in Tamminga (2014), the study of persistence raises new issues for the envelope of variation. Two of these issues are relevant here: the role of the interlocutor and the definition of the variable itself.

**Figure 1:**
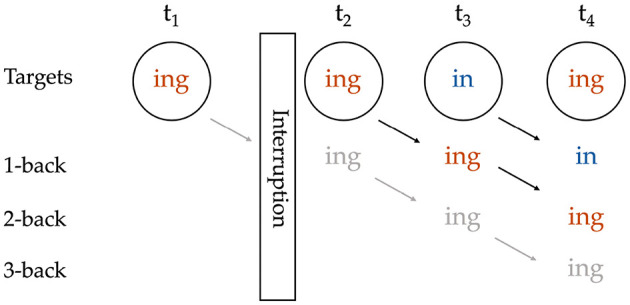
Coding of a sequence with an interruption; grayed-out content reflects potential coding that is blocked by the interruption.

Regarding the role of the interlocutor, in Tamminga (2014, 2016), I omit prime–target pairs that were interrupted by an instance of the variable from an interlocutor. The reason for this decision is that we do not currently know how phenomena like accommodation and interspeaker priming interact with intraspeaker persistence, so we should neither assume that an ING token from an interlocutor is the same as a token from the target speaker and can be included, nor assume that it is irrelevant and can be ignored. Here I extend that decision to the consideration of sequences, making interruption-based exclusions for the length of the N-prior sequence at hand. Figure 1 illustrates that if there had been no interruption, the target at *t*_4_ would have had a 3-prior sequence of /ing-ing-in-T/, while the target at *t*_3_ would also have had a 2-prior sequence of /ing-ing-T/ and could have been included in a 2-prior analysis. But because there is an interruption between the 2-back and 3-back positions relative to the target at *t*_4_, *t*_4_ ends up with no 3-prior sequence, but does still have a valid 2-prior sequence of /ing-in-T/. With this practice, the number of targets that can be included is reduced at each greater depth of prior token sequence.

The second issue is that of the definition of the dependent variable itself. So far I have defined ING as the alternation between the velar and alveolar nasal after unstressed /ɪ/, but complications arise because this alternation occurs in a range of grammatical contexts. Often the ING variable is defined as including progressive verbs and gerunds formed with the *-ing* suffix, such as *working*, monomorphemes like *ceiling*, and the words *something* and *nothing*. However, there has long been uncertainty about whether or not the surface variability in these contexts is the output of a single variable process. In Tamminga (2014, 2016), I show that the monomorphemic (e.g., *ceiling*) and polymorphemic (e.g., *working*) context exhibit within-category, but not across-category, persistence, and argue that this is evidence that multiple variable processes are at play. In this paper, I aim to sidestep rather than illuminate these questions about the definition of the variable. Therefore, I exclude all monomorphemic observations and do not treat them as interruptions because I have already previously shown that they do not influence persistence in the much more frequent polymorphemic cases. On the other hand, in Tamminga (2014) I do find some puzzling evidence for persistence between the polymorphemic categories and *something/nothing*, a category that poses the additional problem of allowing additional variants. I therefore exclude the *something/nothing* category but conservatively treat *something* and *nothing* as interruptions. There is also one other special case, that of the phrase *going to*. I exclude instances of *gonna* from consideration entirely, but treat instances of *going to* that could have been produced as *gonna* as both exclusions and interruptions. Instances of *going to* that could not be realized with *gonna* (such as “I'm going to the store”) are included normally.

At each greater depth of N-prior sequence, some additional data is lost because of interlocutor and exclusion-based interruptions, and additionally the number of unique N-prior sequences increases. There is thus a tension between wishing to look at shorter N-prior sequences because there is more data and a simpler analysis, but also wishing to look at longer N-prior sequences because they provide a more refined view of the time-course of variable production. A 3-prior sequence seems to offer a good compromise between these goals in the particular data at hand, but I also look at the 2-prior and 4-prior sequences. The 2-prior sequence provides a simple starting point for reasoning about sequences of prior observations, and the 4-prior sequence makes it clear that the data at hand should not be stretched further. Overall, approximately the same general pattern arises at the 2-prior, 3-prior, and 4-prior levels, which provides some reassurance regarding the stability of the results.

### 2.3. Descriptive Analysis

**Figure 2:**
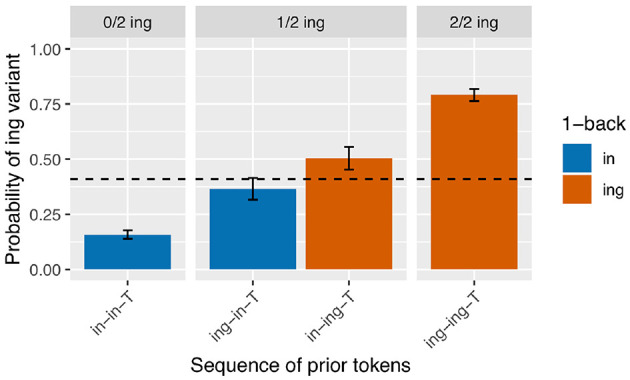
Corpus probability of /ing/ variant by 2-prior sequence. Error bars are Clopper-Pearson binomial 95% confidence intervals.

I begin with an analysis of the subset of the verbal ING data for which the 2-prior sequence is intact (*N* = 3,071). For a depth of two prior observations, there are four unique prior token sequence options: /in-in-T/, /ing-in-T/, /in-ing-T/, /ing-ing-T/ (recall that T represents the linear position of the target). The first two sequences have /in/ as their immediately prior observation, and the last two sequences have /ing/ as their immediately prior observation, so a traditional persistence analysis would group together the first two sequences (as /in/-primed) and the last two sequences (as /ing/-primed). I calculated the /ing/ rate after each of these unique sequences. The results are in Figure 2. The unique 2-prior sequences are arranged on the x-axis, and the y-axis shows the probability of the /ing/ variant after each sequence. To help guide the visual interpretation at the expense of added redundancy, the graph is also faceted by how many /ing/ observations occurred in the 2-prior sequence, and the bars are color coded by the value of the 1-back variant. From Figure 2, it is immediately apparent that the /ing/ rate is higher for observations that had more instances of /ing/ in the 2-prior sequence: the /ing/ rate after two /in/ variants is 16% (*N* = 1,420), while the /ing/ rate after two /ing/ variants is 79% (*N* = 892). In the middle facet of the graph, we see an additional effect: when the 2-prior sequence contains one of each variant, the order they come in matters: the /ing/ rate is higher after an /in-ing-T/ sequence (50%, *N* = 375) than an /ing-in-T/ sequence (36%, *N* = 384). That there is a difference between the two blue bars and between the two red bars in Figure 2 shows that the 2-prior sequence matters beyond supplying the immediate 1-back variant. But that there is a difference between the blue and red bars in the middle facet shows that there is an effect of the 1-back variant that goes beyond the total number of /ing/ observations preceding the target.

**Figure 3:**
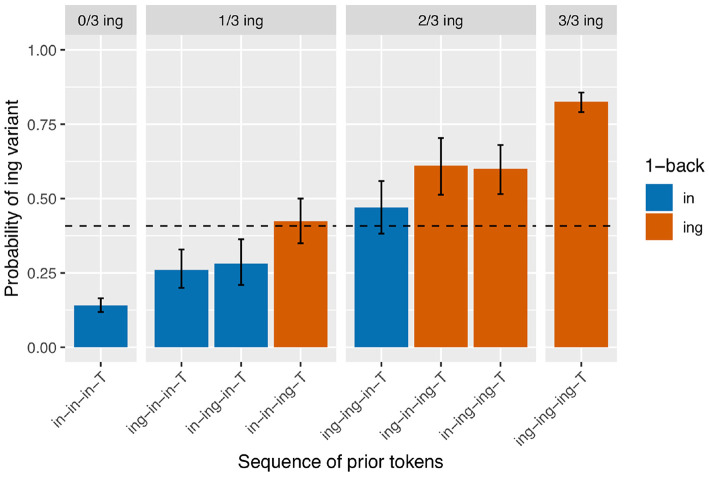
Corpus probability of /ing/ variant by 3-prior sequence. Error bars are Clopper-Pearson binomial 95% confidence intervals.

Next I turn to the subset of the data in which the full 3-prior sequence is intact (*N* = 2,334, so 737 observations removed from the 2-prior subset due to interruptions between the 2-back and 3-back positions). There are eight unique 3-prior sequences to consider, which I will not enumerate here but which can be found listed along the x-axis of Figure 3. Figure 3 is set up in the same way as Figure 2: there is a bar representing the rate of /ing/ use for targets preceded by each of the unique 3-prior sequences, the facets represent the total number of /ing/ variants in the 3-prior sequence, and the color coding represents the 1-back variant. As before, we see a very strong effect at the far ends of the graph: the /ing/ rate after a sequence of three /in/ observations is 14% (*N* = 898) while the /ing/ rate after a sequence of three /ing/ observations is 83% (*N* = 540). In the 1/3 /ing/ facet, we see that the /ing/ rate is higher when the one /ing/ in the sequence is in the 1-back position (42%, *N* = 177) but that the order of the 2-back and 3-back positions does not make a large difference: the /ing/ rate is 26% after /ing-in-in-T/ (*N* = 192) and 28% after /in-ing-in-T/ (*N* = 142) sequences. In the 2/3 /ing/ facet, we see essentially the same thing: the /ing/ rate is depressed when the 1-back token was /in/ (47%, *N* = 132) but does not appear to differ between /ing-in-ing-T/ (61%, *N* = 108) and /in-ing-ing-T/ (60%, *N* = 145) sequences.

**Figure 4:**
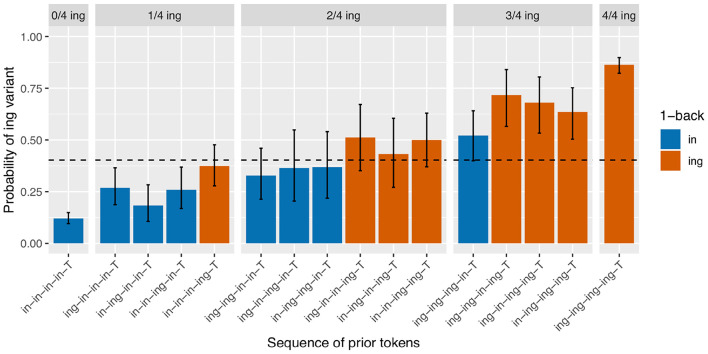
Corpus probability of /ing/ variant by 4-prior sequence. Error bars are Clopper-Pearson binomial 95% confidence intervals.

It should already be apparent from the token counts given in the discussion of the 3-prior sequence results that data sparsity will raise its head as a real problem in the 4-prior sequences, both because there are now 16 unique prior token sequences to subset by and because the total number of observations is down to 1804 after loss of an additional 530 observations due to interruptions between the 3-back and 4-back positions. However, even the smallest subset in this breakdown (/ing-in-ing-in-T/) still has 33 observations in it, so I will cautiously proceed. I will not break down all 16 /ing/ rates shown in Figure 4 in the discussion here, but will instead make some general observations. With less data, the patterns are inevitably somewhat less clear, but there are a couple reasons to believe that the basic result here is consistent with the previous two clearer patterns. First, within each facet, every red bar is taller than every blue bar, and subsequently the average of the red bars is higher than the average of the blue bars across the three middle facets. This is consistent with the observation of an effect of the 1-back variant. Second, within the same-colored bars in each facet, the fluctuations we see are not consistent with plausible predictions from the sequence order. For instance, the /ing/ rate for /ing-in-in-in-T/ is higher than for /in-ing-in-in-T/ even though the latter has a more recent instance of /ing/ in the sequence. This suggests that the deeper-than-1-back order-based fluctuations seen here are random rather than systematic, and that if we had more data in each subset we would expect to see them level out to look more like Figure 3. Of course, the only way to confirm this would be to get more data, a non-trivial task.

### 2.4. Statistical Analysis

In the descriptive analyses just given in section 2.3, I took the following approach at each N-prior sequence depth. First, I calculated /ing/ rates conditioned on each unique N-prior sequence separately. Then, I proposed on the basis of those observed /ing/ rates that treating every unique prior token sequence as a distinct context was missing a generalization: that observed ING rates differ only based on how many /ing/ observations occurred in the prior sequence and what variant is in the 1-back position, not any additional information about the order of variants in the 2-back, 3-back, or 4-back positions. However, the descriptive analyses have not yet accounted for many factors that are known to affect variation in general or ING specifically, such as phonological context or speaker gender. They also do not account for the non-independence that results from different speakers (with different characteristic /ing/ rates) each contributing more than one token to the dataset (prior to the sequence formation process and associated exclusions for interruptions, the average number of observations per speaker is 34). I therefore turn to mixed-effects logistic regression to assess whether the observations I made based on the raw data reflect statistically significant differences that are robust to the inclusion of these other predictors.

The mixed-effects logistic regressions in this section were fit using the lme4 package version 1.1-18 (Bates et al., 2015) in R version 3.5.1 (R Core Team, 2015). The dependent variable is the ING variant in each target observation, with 0 as /in/ and 1 as /ing/. The models include as fixed effects several known predictors of ING that are available in this dataset and were described in Section 2.1, namely lexical frequency, speech rate, preceding segment, following segment, and speaker gender. The lexical frequency measure (Lg10CD) comes from SUBTLEX (Brysbaert and New, 2009) already base-10 log-transformed, and speech rate is natural log transformed. These continuous control predictors are then z-scored to center around their mean log value. The categorical control predictors (preceding/following phonological context and gender) are given a sum-coded (also known as deviation-coded) contrast scheme, so that the intercept in the regression is computed at the grand mean of their levels rather than a reference level. In addition to these fixed effects, each model also includes a speaker random intercept; equivalent models were fit with by-word random intercepts that were dropped because they captured little variance but made generating predicted values more complicated. The speaker random intercept is particularly important, as I discuss in Tamminga (2014), because the non-independence of observations from the same speaker can give rise to apparent “repetitiveness” effects without any true microtemporal clustering involved. Speaker clustering has not yet been controlled out in the mean rates shown in the figures above, so it is crucial to fit these models to account for that non-temporal source of apparent clustering.

I will focus on modeling the 3-prior subset of the data, attempting to capture the pattern seen in Figure 3, rather than modeling the 2-prior or 4-prior sequence analyses. I choose to focus on the 3-prior subset because the 2-prior sequences do not offer enough granularity to look at interesting sequence effects, while the 4-prior sequence analysis has so many prior token sequence conditions that it leaves us without enough data to get a confident probability estimate within each condition. I fit three models to the 3-prior data, which are intended to approximately map to the two-step approach I just recapped for the descriptive data analysis, with Model 1 representing the first step and Models 2 and 3 representing the second step and a refinement thereof. The fixed effects from Model 1 are given in Table 1. Model 1 includes a prior sequence predictor, with a separate level for each unique 3-prior sequence. The levels of this predictor are reverse difference coded, so each level is compared to the previous level. For example, the line in Table 1 labeled “Prior seq. (ing.in.in.T - in.in.in.T)” represents the test of the difference between the probability of /ing/ in an /ing-in-in-T/ sequence and an /in-in-in-T/ sequence. The order of the levels is set to be the same as in Figure 3, so the coefficients in the model represent the difference between the height of each bar and the bar to the left of it (in log-odds). For example, the coefficient for “Prior seq. (ing.in.in.T - in.in.in.T)” maps to the estimated difference between the second blue bar from the left in Figure 3 and the first one on the left.

**Table 1:** Model 1: Each 3-prior sequence compared to the previous 3-prior sequence.

	**Estimate**	**z-value**	**Pr(>|z|)**
Intercept	−0.38	−2.17	0.030
**Control**			
Speech rate	−0.22	−3.41	0.001
Lexical frequency	−0.45	−7.38	<0.001
Preceding coronal	0.28	4.60	<0.001
Following pause	0.35	4.73	<0.001
Female speaker	0.49	2.99	0.003
**Critical**			
Prior seq. (ing.in.in.T - in.in.in.T)	0.12	0.56	0.575
Prior seq. (in.ing.in.T - ing.in.in.T)	0.05	0.19	0.847
Prior seq. (in.in.ing.T - in.ing.in.T)	0.78	2.81	0.005
Prior seq. (ing.ing.in.T - in.in.ing.T)	−0.27	−1.01	0.314
Prior seq. (ing.in.ing.T - ing.ing.in.T)	0.61	2.01	0.045
Prior seq. (in.ing.ing.T - ing.in.ing.T)	−0.16	−0.52	0.602
Prior seq. (ing.ing.ing.T - in.ing.ing.T)	0.59	2.40	0.017

The control predictors are all significant in the expected directions, which is good because they were selected to reflect only known influences on ING. When we turn to the critical predictor of prior sequence in this model, it is important to recall that the contrasts are set up so that each level is compared to the level preceding it. The order of the levels is the same as that in Figure 3: the levels are sorted first by their prior sequence /ing/ count, then by the 1-back position, then the 2-back position, reflecting a plausible expectation that more prior /ing/s might increase the /ing/ rate and, when the number of prior /ing/s is the same, those /ing/s might be expected to be more powerful if they are at a closer sequence position to the target. What we see is that the first three levels do not differ significantly from one another, but then /in-in-ing-T/ significantly favors /ing/ compared to /in-ing-in-T/ (β = 0.78, *p* = 0.005). The next level, /ing-ing-in-T/, does not differ significantly from /in-in-ing-T/, but it is significantly lower than /ing-in-ing-T/ (β = 0.61, *p* = 0.045). The /ing-in-ing-T/ level in turn does not differ significantly from /in-ing-ing-T/. But the final level, /ing-ing-ing-T/, does differ significantly from /in-ing-ing-T/ in favoring /ing/ (β = 0.59, *p* = 0.017). This set of hypothesis tests is consistent with my proposal that there is an influence of the 1-back variant but not deeper (that is, (>1)-back) order effects. The difference tests that are equivalent to the difference between each red bar with a blue bar next to it within a facet in Figure 3—that is, the jump up in /ing/ probability from 1-back = /in/ to 1-back = /ing/, when the prior /ing/ count is the same—show evidence that this 1-back effect is significant. The cases where the 1-back position and the prior /ing/ count are the same do not show evidence for a significant difference. Note that none of these predictors directly test the hypothesis of differences attributable to the prior /ing/ count alone. If there were no prior /ing/ count effect at all, we would expect the comparisons between levels where the 1-back value switches from /ing/ to /in/ but the prior /ing/ count goes up by 1 (as in the comparison between /ing-ing-in-T/ and /in-in-ing-T/ for example) to show a significant decrease in probability (essentially “resetting” back to the blue level instead of the red level). This is not the case. To directly test the idea that there are two things going on, prior /ing/ count and 1-back effect, I will need to fit a model containing those two predictors explicitly. The purpose of Model 1 here is in fact to argue that Model 1 is not the correct model: that in treating every 3-prior sequence as a unique context we are missing a generalization about how the real differences across those sequences can be captured by a pair of overlapping simpler predictors.

Model 2, accordingly, is congruent with that proposal: instead of a single predictor with a different level for each prior token sequence, I include two predictors, one for /ing/ count in the prior sequence (the equivalent of the facets in Figure 3) and one for the 1-back variant (the equivalent of the bar colors in Figure 3). The prior /ing/ count is treated as a categorical predictor here, again using reverse difference coding for the contrasts. The results from this model are given in Table 2. There is a significant effect such that if the 1-back variant is /ing/, the target is more likely to be /ing/ (β = 0.68, *p* <0.001). While the size of the coefficient is quite similar to the comparisons in Model 1 that amounted to a test of a 1-back effect while controlling prior /ing/ count (which were 0.78 and 0.61), pooling over all of the prior /ing/ count values approximately doubles the effect size (z). When we look at the prior /ing/ count predictor, we can see that the difference between 1 and 0 prior /ing/s is not significant but all other comparisons between levels are. This is consistent with what we saw in Model 1 with the lack of difference between the first two levels of the prior sequence predictor.

**Table 2:** Model 2: Categorical prior /ing/ count and 1-back.

	**Estimate**	**z-value**	**Pr(>|z|)**
Intercept	−0.68	−3.62	<0.001
**Control**			
Speech rate	−0.22	−3.46	0.001
Lexical frequency	−0.45	−7.35	<0.001
Preceding coronal	0.28	4.58	<0.001
Following pause	0.35	4.72	<0.001
Female speaker	0.49	2.99	0.003
**Critical**			
1-back /ing/	0.68	4.07	<0.001
Prior /ing/ count (1-0)	0.20	1.12	0.262
Prior /ing/ count (2-1)	0.38	2.15	0.032
Prior /ing/ count (3-2)	0.47	2.32	0.020

Model 3 reflects a refinement of Model 2 but keeps the basic premise of the model. The only difference between Model 2 and Model 3 is that Model 3 treats prior /ing/ count as a continuous numeric predictor instead of a categorical predictor. In one sense this is not the correct thing to do: an integer count value is a different sort of thing than a continuous number, and the only options for prior /ing/ count values are integers. However, what it reflects in this model is the premise that what we're trying to capture with the prior /ing/ count predictor is something like “how /ing/-ful is the speaker's overall recent prior experience,” and we only have a coarse-grained measure of what is underlyingly a continuous measure. In theory we might want to look at something like a weighted moving average over a larger window to get a more truly continuous measure of “how /ing/-ful is the speaker's overall recent prior experience.” The reason I do not undertake such an analysis is that the problem of interlocutor interruptions makes it difficult to go very far back. In any case, Table 3 presents the results of Model 3. It shows that the 1-back estimate is stable but now the linear prior /ing/ count predictor has a larger effect size and much smaller *p*-value than any of the corresponding prior /ing/ count values in Model 2.

**Table 3:** Model 3: Continuous prior /ing/ count and 1-back.

	**Estimate**	**z-value**	**Pr(>|z|)**
Intercept	−1.19	−6.38	<0.001
**Control**			
Speech rate	−0.22	−3.50	<0.001
Lexical frequency	−0.45	−7.31	<0.001
Preceding coronal	0.28	4.60	<0.001
Following pause	0.35	4.69	<0.001
Female speaker	0.49	3.03	0.002
**Critical**			
1-back /ing/	0.67	4.06	<0.001
Prior /ing/ count	0.35	3.79	<0.001

The three models I have fit here are not nested, and therefore cannot appropriately be compared formally with log-likelihood tests. However, various model criteria might support an informal comparison of the models. Each model is simpler than the last in terms of degrees of freedom (Model 1 d.f. = 14, Model 2 d.f. = 11, Model 3 d.f. = 9). As a result, the log likelihood inevitably goes up, but only slightly: the log likelihoods of the three models are −1058.2, −1058.8, and −1059.4, respectively. Meanwhile, the AIC and BIC measures, which penalize extra parameters, go down from Model 1 (AIC = 2144.5, BIC = 2225.1) to Model 2 (AIC = 2139.5, BIC = 2202.8) and from Model 2 to Model 3 (AIC = 2136.8, BIC = 2188.6). These criteria are in line with the view that Model 3 is the simplest and strongest model of the prior sequence effects in this data.

**Figure 5:**
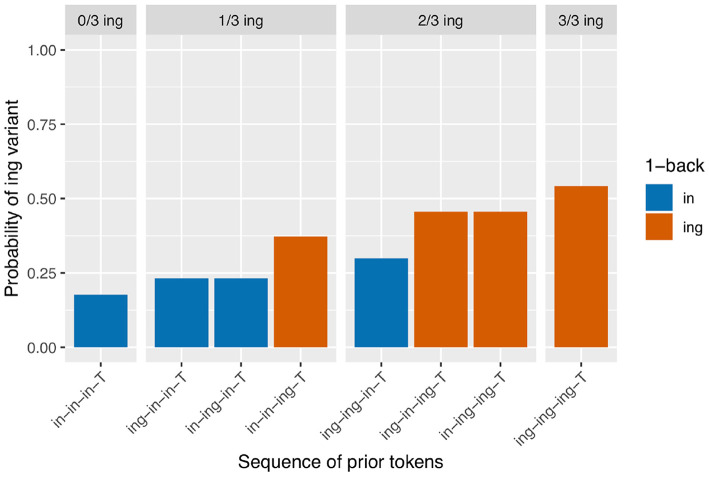
Predicted values from Model 3 (male speaker PH06-2-2 with observed /ing/ probability = 0.4, non-pre-coronal, non-post-pausal, scaled log vowels per second = 0, scaled Lg10CD = 0).

Figure 5 shows a data visualization that is equivalent to the observed data visualization in Figure 3 but instead represents the predicted probabilities from Model 3 for a particular male speaker (PNC PH06-2-2) whose mean /ing/ rate is near the dataset grand mean, for a token that neither follows a coronal nor precedes a pause and has a scaled log vowels per second of 0 and a scaled Lg10CD value of 0. This illustrates that this model is producing predictions that are a good match for the empirical patterns we saw in section 2.3—these patterns remain when we control for speech rate, frequency, phonological context, speaker gender, and speaker identity clustering.

## 3. Prior Sequences in Simulated Data

The empirical data in section 2.3 showed the same pattern at three lengths of N-prior sequence: the probability of /ing/ at a target is affected by both the total number of /ing/ instances in the N-prior sequence and the variant used at the 1-back position (that is, the token that would normally be treated as the prime), without evidence to suggest that it is influenced by the order of prior observations at an N-back position of N greater than 1. The statistical modeling in section 2.4 supported that interpretation of the data while controlling for other known predictors of ING. But what does this result actually tell us about the source of persistence? In this section I aim to show that this type of analysis can move us toward an answer on a problem that has seemed intractable for some time.

In this section I use a series of simple Bernoulli process simulations to explore the potential processes generating different patterns of target probabilities based on prior token sequences. It should be emphasized that this is a preliminary tour through what I believe could become a fruitful area of research more broadly. The use of computational simulations in sociolinguistics is not new, but most simulations are simulations of communities, such as agent-based models of the spread of sound change through a population over generations. The simulations I use here are focused on a microtemporal level and are conceptually very simple: I model the production of variation essentially as strings of coin flips at different probabilities, then analyze the generated data in the same way as I analyzed the corpus ING data. I compare the output of different simulated models to the corpus results from the previous section as a way of investigating the plausibility of different processes having generated the data. I particularly pay attention to the ways in which the predictions from models of baseline deflection and models of sequential dependence are dissociated under various conditions. This is of interest because it motivates the study of multiple token sequences in contrast to the usual persistence approach (looking at only one prior token) that does not distinguish between baseline deflection and sequential dependence. While I will not be able to conduct an exhaustive search of the many-dimensional parameter space opened up by these models, my preliminary explorations here will suggest that a model combining both a sequential dependence mechanism and a baseline deflection mechanism produces patterns that most closely and consistently resemble the results of the corpus data analysis in section 2.3.

### 3.1. Simulation Preliminaries

For clarity of exposition with a sociolinguistic audience in mind, I will discuss the models here as if they involved speakers producing the ING variable: for instance, I will describe a Bernoulli trial^9^ with an outcome of 1 as an instance of the /ing/ variant. I will also present visualizations of the model outputs using this framing around ING, making the graphs directly visually comparable to the graphs in section 2.3. It should, of course, be borne in mind that everything happening in these simulations is merely lists of probabilities and 0s and 1s; nothing about them is specific to ING (or to sociolinguistic variation, or, indeed, to linguistic behavior).

**Figure 6:**
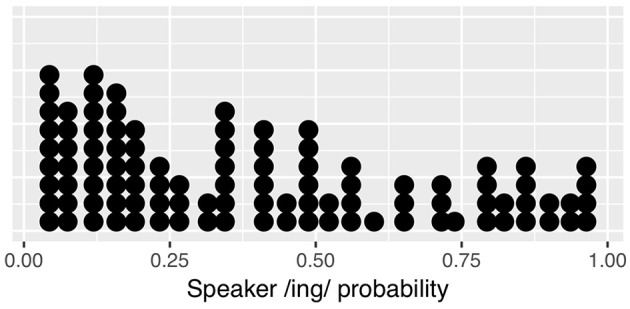
Observed by-speaker probabilities from corpus data, used for simulated speaker baselines.

Each simulation involves the same set of simulated “speakers,” whose identity is tracked during each run of the simulation. Each speaker has some baseline probability of producing the /ing/ variant (vs. the /in/ variant). These baseline probabilities are taken from the observed corpus data so that the overall distribution of speakers and their linguistic behavior resembles that of the real data. In the corpus 3-prior dataset, there are 118 speakers who each produce on average 34 observations. Of these, 17 speakers end up contributing only /ing/ or only /in/ outcomes to the 3-prior data, but only because of exclusions: none of these are speakers whose ING behavior is categorical in the larger data set. However, in the interest of avoiding simulated speakers with categorical baselines, I exclude these 17 speakers in order to end up with 101 simulated speakers with non-categorical baselines. The distribution of by-speaker baseline /ing/ probabilities is shown in Figure 6. Each of the simulated speakers will produce an ordered string of 20 “ING tokens” (Bernoulli trials) with the speaker's /ing/ probability as the outcome probability of each trial. Since the first three trials from each speaker are excluded from analysis because they do not have enough previous trials, each speaker contributes 17 observations to the simulated data set, resulting in a total of 1717 observations in each simulated data set (compared to 2300 in the observed data at 3-prior depth). I calculate the observed proportion of 0s and 1s conditioned on each preceding trial sequence, then store these values. The entire run is then repeated 500 times and the distribution of results from those runs is presented graphically. I also fit a linear mixed effects regression to each simulation run, with predictors equivalent to the critical predictors from Model 3 from the corpus data analysis plus the speaker random effect (the control predictors in Model 3 are not relevant for the simulated data). I extract the 1-back and prior /ing/ count predictor z-values (effect sizes) and p-values from each run over the course of the 500 runs in order to find out how often each simulation produces statistically significant effects aligned with the corpus results.

The series of simulations that I will compare across the following subsections is built up as follows. The first simulation, in section 3.2, contains no microtemporal clustering: I call this the null simulation. Each subsequent simulation has some source of microtemporal clustering added in. In the sequential dependence simulations in section 3.3, the built-in clustering mechanism that is added to the null simulation is that the outcome of each trial affects the outcome probability for the next trial. In the baseline deflection simulations in section 3.4, a different built-in clustering mechanism is added to the null simulation: each speaker has two or more states with distinct target probabilities that are above and below the speaker's characteristic probability. These create the possibility of baseline deflection as the speaker moves between different states and thus different target probabilities; a Markov chain model generates the sequences of states that the speakers move through. Finally, in section 3.5, both of these distinct clustering mechanisms are included in the simulation at the same time. In all simulations, the data is generated by sampling the binomial distribution randomly at each trial (at the specified probabilities) using the binom package in R.

### 3.2. The “Null” Simulation: No Microtemporal Clustering

The first thing I do is show what the N-prior sequence effects look like in data that has speaker clustering (speakers differ in their characteristic rates) but no form of microtemporal clustering (that is, neither sequential dependence nor baseline deflection, with no intraspeaker probability fluctuation). I call this the “null” simulation because of the lack of critical clustering structure. This simulation is important because it would be easy to mistake speaker clustering for within-speaker temporal structure. This will also be a starting point for the creation of various microtemporally structured probability patterns that I will use in the subsequent simulations.

**Figure 7:**
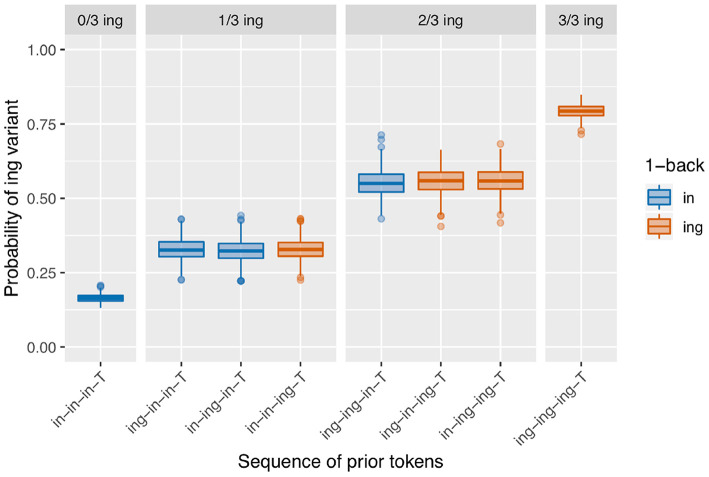
Simulation with speaker baseline differences but no built-in microtemporal clustering.

The speaker baselines in the null simulation are as just discussed in section 3.1 and shown in Figure 6. The results of the null simulation are shown in Figure 7. What is immediately apparent is that the effect of the prior /ing/ count seen in section 2.3 arises from speaker clustering without any within-speaker microtemporal structure. This makes sense: without controlling for speaker clustering, a target preceded by three /ing/ outcomes is more likely to be a target from a high-/ing/ speaker and therefore more likely to itself have an /ing/ outcome. While there would be an apparent 1-back effect if we looked only at the 1-back prior token depth (the red boxes are on average higher than the blue boxes), we do not see any 1-back effect beyond that generated by the prior /ing/ count, which is also as expected. The regression results from the simulations confirm that the 1-back effect is not present (a significant positive effect on 1.8% of trials and a significant negative effect on 2.8% of trials).

In theory, including random speaker intercepts in a linear mixed effects model of each simulation's data should eliminate the visually-apparent /ing/ count effect. The statistical model values show that actually the models end up somewhat anti-conservative: there is a significant positive effect of prior /ing/ count on 11.4% of runs. Because the structure of the model does not include any possible true microtemporal source of this effect, we can be confident that these findings actually arise from incompletely controlled speaker clustering ^10^. This should be kept in mind when interpreting the other models; I will compare the observed number of significant prior /ing/ count effects to this rate ^11^.

### 3.3. Simulating Sequential Dependence

I now build on the null simulation by adding the first candidate source of within-speaker microtemporal structure: sequential dependence. This simulation is identical to the previous one except that, within each speaker, the outcome probability of each Bernoulli trial is slightly influenced by the outcome of the previous trial. I set the probability adjustment to 0.05: if the prior outcome was a 1, I add 0.05 (out of 1) to the target probability, and if the prior outcome was a 0, I subtract 0.05 from the target probability. The probability adjustment is always done to the speaker's base probability, so the probabilities don't snowball and go out of bounds. Notice that this is equivalent to each speaker having two states with different /ing/ probabilities, with the state they are in on each trial determined by the ING outcome of the previous trial. Any number of more sophisticated adjustments to the baseline could be used to generate the exact /ing/ probabilities for these states; the ± 0.05 adjustment is simple and transparent but is not intended to involve any substantive claim about how these probabilities are or should be adjusted.

**Figure 8:**
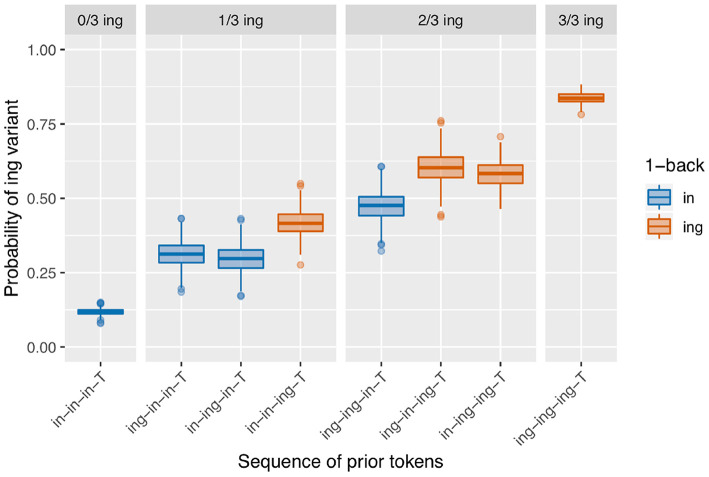
Results of 500 runs of a sequential dependence simulation with a 0.05 boost.

The results we see in Figure 8 bear a resemblance to the observed data in Figure 3. We see what looks like the prior /ing/ count effect, although the null simulation made it clear that this can derive from speaker-level clustering. We also see an effect where the red boxes are higher than the blue boxes within each facet: the only-1-back effect. This model produces a significant positive 1-back effect on 73.4% of runs, but a significant positive prior /ing/ count effect only 8.6% of the time—the latter being slightly lower than the false positive rate in the null simulation. In other words, all of the apparent /ing/ count effect here is attributable to the speaker rather than temporal clustering. Interestingly, there is also a small difference between the /ing-in-in-T/ and /in-ing-in-T/ conditions in the 1/3 ing facet, and between the /ing-in-ing-T/ and /in-ing-ing-T/ conditions in the 2/3 ing facet. These differences result from small biases in which types of speakers produce which prior token sequences ^12^. Consider the 2/3 /ing/ sequences. If a speaker has a low /ing/ baseline probability, they are slightly more likely to produce an /ing/ after another /ing/ (as in /in-ing-ing-T/ due to the facilitating effect of the first /ing/) but less likely to spontaneously produce /ing/ twice apart from that facilitating influence, as in /ing-in-ing-T/. In contrast, it is somewhat “easier” for a high-/ing/ speaker to produce the two /ing/s spontaneously. As a result, /ing-in-ing-T/ prior sequences are slightly more likely to come from high-/ing/ speakers, and subsequently slightly more likely to result in an /ing/ outcome.

### 3.4. Simulating Baseline Deflection

In the next set of simulations, I investigate baseline deflection instead of sequential dependence. I remove from the simulations the mechanism of adjusting the target probability based on the prior outcome. Instead, I give each speaker two target probabilities that average to the same characteristic probability as they had in the previous simulations, when possible. Specifically, I add and subtract 0.3 from the baseline, so for example a speaker with an overall baseline of 0.4 will have a state A /ing/ probability of 0.1 and a State B /ing/ probability of 0.7. When this calculation would put the probabilities outside of the 0 to 1 range, I replace the value with 0 or 1 accordingly—so, speakers can have a categorical behavior in one of their two states. The speaker then switches back and forth between states A and B over stretches of trials.

The state-switching behavior in the simulation is generated stochastically using a Markov process: each state has a transition probability reflecting the likelihood that the process will switch to the other state for the next trial, but there is no further time dependence. I use symmetrical transition probabilities throughout the simulations I present here (so the probability of switching from A to B is the same as the probability of switching from B to A) but will present several different transition probabilities reflecting different degrees of state “stickiness.” The use of the Markov process to generate the state switches is not intended as a claim that this kind of state switching is actually generated stochastically. On the contrary: I expect that changes in state would reflect responses to changes in the real world context where the speech is taking place, such as changes in topic, context, or interlocutor, or changes in the speaker's internal state, such as shifts in stance, attitude, or attention. From the perspective of the analyst, however, such contextual changes are unpredictable and therefore can be modeled as a stochastic process ^13^. Once the sequence of states has been determined, there is a Bernoulli trial with the probability of success equal to the output probability at each trial's predetermined state, which produces the /ing/ or /in/ variant as in the previous simulations. The idea is to produce a model capturing the intuition that when two trials are closer together they are more likely to be in the same state, and therefore more likely to have the same outcome. The most important property of the model is simply that the state sequences are generated independent of the outcomes at each trial.

This approach to the simulation of baseline deflection offers different parameters that could be adjusted to generate a very wide range of possible outcomes. Here I present versions of the simulation at four different between-state transition probabilities. I do not change any other parameters: I hold the number of states (two) and the size of the difference between them for each speaker constant and do not allow for one state to be stickier than the other or for the stickiness of states to change over time.

**Figure 9:**
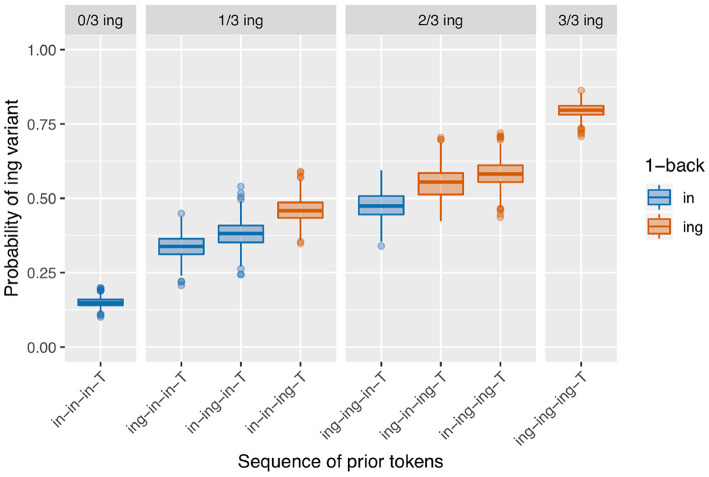
Results of 500 runs of a baseline deflection model with between-state transition probability of 0.1.

When the transition probability is low, so the states are quite sticky, the result is a pattern that reflects the continuous effect of a prior token sequence such that the more prior /ing/s there are, and the closer in the sequence they are to the target, the higher the observed /ing/ rate in the target will be. This is shown in Figure 9 for a model where the transition probability out of both states is 10%. I call this a continuous-N-back effect, in contrast to an only-1-back effect. In the regression models extracted over the runs of the simulation, this simulation produces a significant positive /ing/ count effect on 99.8% of runs, and a significant positive 1-back effect on 71% of runs. This seems promising, but recall that the model is not actually set up to detect a difference between a continuous-N-back effect and an only-1-back effect; visual inspection of the output in Figure 9 suggests that this is a somewhat different pattern than what we see in the corpus data. In a model where the transition probability is 50% for both states, so speakers are equally likely to stay in their current state or switch to the other state, then both the 1-back and prior /ing/ count effects are lost: there is a significant positive /ing/ count effect on 10.8% of runs, again comparable to the null rate, and a significant positive 1-back effect on 1.4% of runs. The output of the model is not shown here but is visually identical to that of the null model.

**Figure 10:**
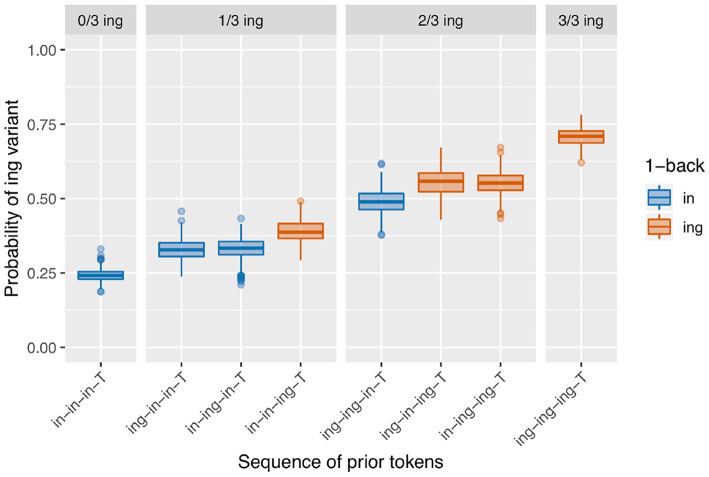
Results of 500 runs of a baseline deflection model with between-state transition probability of 0.4.

It is also possible to get a result that looks like the 1-back result from the sequential dependence model. This arises when the transition probability for both states is just shy of 50%, so a speaker is a little more likely to stay in their current state than not: Figure 10 shows the results when the transition probability is 40%. This model produces a significant positive 1-back effect on 36.6% of runs, which is not trivial but also not as good as the sequential dependence model where 73.4% of runs produce a 1-back effect. Like the sequential dependence model, though, this simulation mostly loses the significant prior /ing/ count effect, producing a significant positive /ing/ count effect on only 17.8% of runs, not a very big improvement over the 11.4% positive results in the null simulation.

**Figure 11:**
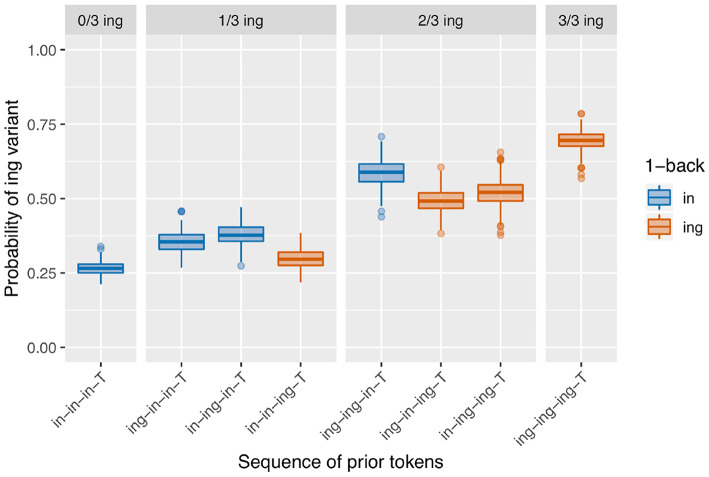
Results of 500 runs of a baseline deflection model with between-state transition probability of 0.6.

Interestingly, these simulations are also able to reverse the direction of at least the 1-back pattern. Figure 11 shows that as soon as the transition probability in each state is over 50%, the direction of the 1-back effect reverses, so that at each value of the prior token count, the contexts where the prior /ing/s were further away have the higher /ing/ probability, which is not as we would generally expect given the usual persistence pattern. The statistical models confirm this reversal: on 67.6% of runs of this simulation there is a significant negative 1-back effect. This reversal reflects the fact that when the transition probability is over 50%, two sequentially adjacent tokens are actually *less* likely to occur in the same state, rather than more likely, because from token to token the state is more likely to switch than to stay the same. This highlights that the argument in favor of baseline deflection as a source of repetitiveness does contain some assumptions about the time course of baseline deflection, namely that the window over which the baseline might shift is sufficiently wide that in fact two tokens occurring sequentially are more likely to be produced in the same window than not. It is also worth noting that there is an attested pattern of anti-persistence in the literature, which Szmrecsanyi (2006) terms the *horror aequi* effect. This particular simulation gives us one way of understanding how such an effect could arise.

### 3.5. Combining Sequential Dependence and Baseline Deflection

Both of the simulation types discussed so far have drawbacks in terms of the likelihood that their microtemporal clustering model might have produced the corpus ING data discussed in section 2.3. The sequential dependence model nicely produces an only-1-back effect reminiscent of the distinct pattern seen in the corpus data, but produces a prior /ing/ count effect only at chance rates. The baseline deflection models can clearly produce a wide range of patterns. But in the case where a baseline deflection model does consistently give rise to the desired prior /ing/ count effect (the version with the lowest transition probability), it also produces a continuous-1-back pattern rather than an only-1-back pattern.

**Figure 12:**
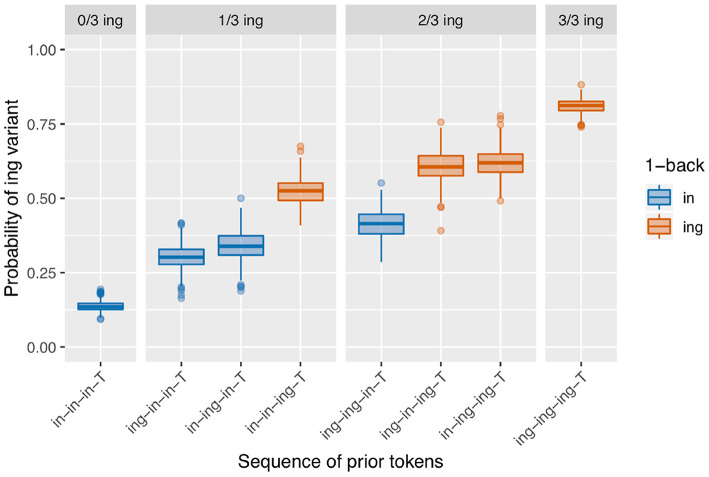
Model with both baseline deflection (transition probability = 0.1) and sequential dependence (boost = 0.05).

There are two model classes under consideration here, and two empirical effects we desire to produce with the models. It seems that each model is better suited to producing one of the empirical effects: most versions of the baseline deflection models produce an /ing/ count effect, and the sequential dependence model produces an only-1-back effect. An appealing next step, then, is to combine the models to create a simulation that has both sequential dependence and baseline deflection built in. In this simulation, the state-shifting behavior is first generated using a Markov process as in the baseline deflection models; then the coin-flipping procedure takes place with the sequential dependence boosting behavior built in. The results of a set of simulations of this type with transition probability of 0.1 (as in the baseline deflection model of Figure 9) and a boost of 0.05 (as in the sequential dependence model of Figure 8) are shown in Figure 12.

This set of simulations now has several desirable features. The basic pattern of results shown in the graph more closely resembles an only-1-back effect than a continuous-1-back effect, making it an improvement over the component baseline deflection model alone; this is achieved through the inclusion of the sequential dependence boost. In terms of the model fit, we get a significant /ing/ count term on 99.2% of runs and a significant 1-back term on 99.6% of runs. By combining these two sources of microtemporal clustering into a single model—in a way that is consistent with the existence of multiple independently motivated phenomena that we expect to shape linguistic behavior in speech—we are able to more consistently arrive at an outcome that resembles the corpus data.

## 4. Discussion

The sizable corpus sociolinguistic literature on persistence has typically asked how a single prior instance of a variable affects the outcome in a target instance of the same variable. In the first part of this paper, I extended this view of persistence to ask what effect sequences of multiple prior tokens have on the outcome of a target token. The descriptive results in section 2.3 indicate that this analysis of sociolinguistic sequences can reveal additional microtemporal structure that is not visible when we look only at a single prior token. More specifically, there are two aspects of the corpus ING results that are of interest and would not be detectable with the 1-back information only. First, there is a cumulative effect of how many /ing/ tokens occur in the prior token sequence, regardless of their position. This effect goes beyond the clustering we expect merely from differing speaker baselines. Second, there is a distinct effect of what variant occurred in the 1-back position. If we look only at the previous token, we would not be able to see either effect: we could not tell the difference between 1/3 and 2/3 of the prior tokens being /ing/ if we had only one token, nor would we be able to tell that the order of previous tokens is irrelevant beyond the 1-back position.

In the second part of the paper, I have suggested that this enriched view of the microtemporal structure of sociolinguistic repetitiveness can bring new evidence to a longstanding debate about the nature of that repetitiveness. The observation of persistence in corpus data has often been interpreted as reflecting sequential dependence, where the outcome of a prior instance of the variable directly influences the target outcome. On the other hand, it is often objected that persistence might arise as a result of baseline deflection, where sequential tokens are more likely to occur under similar contextual circumstances and therefore more likely to have the same outcome. To clarify what these two types of microtemporal clustering predict, I built a number of simulations in which sociolinguistic variation between /ing/ and /in/ is modeled using Bernoulli processes. In these simulations, sequential dependence is modeled by allowing the outcome of one Bernoulli trial to adjust the outcome probability on the next Bernoulli trial, while baseline deflection is modeled by creating pre-established sequences of states with different outcome probabilities but then not making reference to the actual *outcomes* across trials.

The sequential dependence model produces one of the two central effects of interest in the empirical data, the only-1-back pattern (seen in Figure 8). From a mechanical point of view, this can be understood straightforwardly: the sequential dependence models were built such that the target trial is only given information about the outcome of the immediately prior trial, not of previous trials. Of course, nothing would prevent us from building a sequential dependence model that adjusts the target trial probability based on the outcome information from several previous trials. The corpus result, then, is not trivial; the usefulness of a sequential dependence model that only tracks a single prior token suggests that it may be worthwhile to investigate comparable real-world processes that operate over long distances in terms of time yet a limited window in terms of prior instances of the linguistic variable. A downside of the sequential dependence model is that it does not reliably produce the /ing/ count effect. It is possible to build a baseline deflection model that mimics the output of this sequential dependence model (as in Figure 10), but such a model ends up with the same drawback as the sequential dependence model in that it also does not reliably produce the /ing/ count effect. On the other hand, a baseline deflection model with a relatively low between-states transition probability of 0.1 has the advantage of almost always producing a significant /ing/ count effect as desired. However, it does not produce the same kind of separation between 1-back (and only 1-back) conditions as the corpus data exhibits. Instead, it produces a continuous effect of recent /ing/ tokens: the more /ing/s and the closer those /ings/ in the prior token sequence, the greater the likelihood of /ing/ in the target (as seen in Figure 9). While we might have expected such a continuous-N-back effect on intuitive grounds, it does not actually accord with the pattern seen in the corpus data. In section 3.5, I showed that combining the sequential dependence and baseline deflection clustering mechanisms into a single model produces a surface pattern that is a near match for the corpus data, as well as nearly-always significant critical main effects from the regression models.

That the combined simulation seems to most successfully match the corpus data is an appealing result because we have independent evidence for the real-world phenomena that might produce both types of microtemporal clustering. As I discussed in section 1.1, there are multiple candidate phenomena that might give rise to each of the two types of microtemporal clustering under consideration here. Priming is the most commonly appealed-to phenomenon generating sequential dependence, but other sources of true sequential dependence are possible. Style shifting, broadly construed, is the most frequently suggested phenomenon that could give rise to co-occurrence through baseline deflection. To reiterate the point in section 1.1, nothing in this paper should be taken as evidence for or against particular mappings of clustering types to real-world interpretations. However, the fact that phenomena that could produce both clustering types unquestionably *exist* means that a model in which multiple phenomena are at play is an entirely plausible one. For example, were we to think that baseline deflection arises from contextual style-shifting while sequential dependence arises from priming of a recently-used linguistic option, we might find it entirely unsurprising that speakers are both style-shifting and exhibiting priming at the same time: there is plenty of evidence for the existence of both style-shifting and priming in human linguistic behavior. Indeed, to conclude that one of those phenomena was not at play might be even more surprising. The same logic applies to other possible interpretations of the sources of microtemporal clustering; the current study has nothing to say about where sequential dependence and baseline deflection come from, although conceivably some outgrowth of this approach could be used to probe for more precise quantitative properties of priming and style-shifting in future work.

Of course, the analyses and results of this paper are far from conclusive; they are best treated as a promising methodological demonstration inviting further research. One possibility that should be kept in mind is that the particular properties of the corpus results themselves could have occurred by chance. I have explored the simulations with a view to identifying a model that could plausibly have generated the corpus results as observed. But given the role of chance as well as possible uncontrolled factors in conversational speech data, one possibility is that the corpus results themselves are a chance output of a model like one of the models I have deemed less successful. Even if the pattern of results seen here is not due to chance, it might still be true that the pattern reflects something specific about the particular conversational interactions in the PNC data, or something unique to Philadelphia English, or something about the ING variable itself. We should be cautious to not reify or over-interpret the “prior /ing/ count” and “only-1-back” effects as I have described them here. The basic persistence effect has been found repeatedly across many different studies and therefore is seen as demanding a relatively general explanation; no deep investment in general explanations of these longer sequence effects should be made unless they can also be established as more generally recurring properties of sociolinguistic sequences. The most important step toward building confidence in this pattern of results will be to repeat the analysis on other ING data sets, other English variables besides ING, and ideally other languages entirely.

There are also many possible analyses that this paper has not undertaken. My preliminary explorations of the simulations have barely broached the many-dimensional parameter space afforded even by the simple models used here. Furthermore, the models could be enriched in many ways. While it would probably not be useful to simulate all of the possible details of ING variation simultaneously, one particular factor that has not played a role in any of the analyses thus far is the amount of time that elapses between each token. In previous work I have shown that the decay of ING persistence is very slow (Tamminga, 2014), which suggests that decay is unlikely to play a major modulating role in the effects we see when we abstract away from the exact duration of the time between a prior token and a target. An additional practical consideration in omitting temporal lag as a factor in the corpus analysis is that it is not, at first glance, obvious how best to combine the different prior token sequences with all of the possible decay relationships between them. However, future work might explore ways of integrating a continuous time dimension into the analysis of prior token sequences.

The goal of this paper was to show that there is value in the study of sociolinguistic sequences and the microtemporal structure they reveal. Sequential dependence and baseline deflection seemed inextricably intertwined in the 1-prior view, and indeed every single simulation in section 3 produces an overall difference between 1-prior conditions that would be counted as a finding of persistence under traditional quantitative approaches to persistence. Through the simulations, though, we learned that a longer time window can give us a more nuanced picture of what speaker repetitiveness looks like, with baseline deflection and sequential dependence producing outcomes that can be seen to be different when we look at longer prior sequences. We have already made much progress through the study of persistence at the 1-prior depth; as Szmrecsanyi concludes, “persistence is actually sufficiently patterned and predictable to help us understand better the linguistic choices that speakers make” (Szmrecsanyi, 2006, p. 6). The combined corpus analysis and simulations here suggest that this sentiment is as true of longer sequences as it is of prime–target pairs. The potential in modeling longer sequences can be seen from this study regardless of whether the particular analyses offered here are correct. We have not yet reached the limits of what we can learn using persistence, 1-back or N-back, as a tool for the investigation of sociolinguistic variation. By investigating quantitative patterns at the microtemporal level, we can learn more about what factors are at play in the production of sociolinguistic variation.

## Data Availability

The datasets generated for this study are available on request to the corresponding author.

## Author Contributions

The author confirms being the sole contributor of this work and has approved it for publication.

### Conflict of Interest Statement

The author declares that the research was conducted in the absence of any commercial or financial relationships that could be construed as a potential conflict of interest.

## Footnotes

^1^It has also sometimes been called perseverance, perseveration, serialism, parallelism, and most colorfully, the “birds of a feather” effect.

^2^Priming itself might arise from a variety of mechanisms, such as spreading activation or error-driven implicit learning, any of which would fall under the umbrella of sequential dependence.

^3^Or more, although I will not consider variables with more than two variants here.

^4^I explicitly include the T in the sequence name to make the directionality clear.

^5^Note that in these cases, the hyphens elide an unknown amount of speech between observations of the variable; in section 4 I will briefly address the question of the distance between observations, but I will mostly leave modeling of decay in multi-token sequences for later work.

^6^Following one variationist convention, the all-capitalized representation ING represents the variable itself, the choice between two outcome variants.

^7^I will use orthographic representations inside slashes for the variants: /ing/ for /ɪŋ/ and /in/ for /ɪn/ to achieve consistency with my sequence notation. For N-prior sequences, I put the entire sequence between a single pair of slashes. In graphs, I omit the slashes as unnecessary visual clutter. Although unconventional, I believe this is the most visually distinctive set of options, and therefore is to be preferred as a way of making the complex discussion slightly easier to follow.

^8^Thanks to Joe Fruehwald for sharing his handCoder.Praat script.

^9^A Bernoulli trial is simply a random variable with only two possible outcomes, sometimes treated as “success” and “failure.” The probability of success and probability of failure add up to 100%. A familiar example of a Bernoulli trial is a coin flip.

^10^A “true” null simulation would be one that simply contains 1717 Bernoulli trials at a single probability, which should produce a spurious prior /ing/ count effect only 5% of the time.

^11^Of course, the empirical data should also be reassessed in light of this finding, but because the prior /ing/ count *p*-value from Model 3 is very low, I will continue with the assumption that this effect is unlikely to be due to chance even with the elevated probability of a spurious result.

^12^Thanks to Dan Lassiter for identifying the source of these differences.

^13^Thanks to Kory Johnson for this suggestion and for proposing the use of Markov processes for this purpose.
